# Reply to Comment by Young *et al*.

**DOI:** 10.1098/rspb.2008.1781

**Published:** 2009-01-13

**Authors:** F. Mathews, P. Johnson, A. Neil

**Affiliations:** 1 School of Biosciences, University of ExeterExeter, Devon EX4 4QJ, UK; 2 WildCRU, Department of Zoology, University of OxfordTubney House, Tubney OX13 5QL, UK; 3 Division of Public Health and Primary Health Care, University of OxfordOxford OX3 7LF, UK

Had our study been, as Young *et al*. imply, a data trawling exercise using hundreds of variables to look for a ‘thread’, then doubts about its validity might be justified. However, their account of our work bears little relationship to the methods, results or conclusions we report. For example, Young *et al*. claim that we used 396 tests to address our primary hypothesis. In fact, we used two.

Young *et al*. fail to acknowledge that our work was based on a strong *a priori* hypothesis. Far from being ‘genetically implausible’, it has long been accepted that human sex ratios at birth are not 50 : 50 despite the equal production of ‘male’ and ‘female’ sperm, and that birth sex ratios vary between populations and across time. These facts have provoked debate about the likely role of the parental environment in influencing infant gender (Krakow 1994; Lazarus 2002; Rosenfeld & Roberts 2004; Sheldon & West 2004; Wild & West 2007). Our primary hypothesis, based on sound evolutionary principles and supported by considerable research on other mammal species, was that women with good nutrition at the time of conception would be more likely to bear sons. Even since the publication of our paper, two further papers on humans have appeared supporting this hypothesis (Bulik *et al*. 2008; Villamor *et al*. 2008).

The ‘main finding’ of our study (notwithstanding Young *et al*.'s preoccupation with cereal) is a link between maternal nutritional status around conception and infant gender. Because intakes of different nutrients are inevitably correlated with one another (people eat food, not single nutrients), we used a standard method of data reduction—principal components analysis (PCA)—to summarize the patterns of nutrient intakes along new axes (‘components’). PCA is widely advocated as a means of dealing with collinearity (the non-independence of predictors) that would otherwise violate one of the fundamental assumptions of regression analysis (see Massy 1965; Feinstein 1996; Glantz & Slinker 2001; Grafen & Hails 2002; Zuur *et al*. 2007). Its use made it unnecessary to conduct multiple tests on individual nutrients to examine the primary hypothesis of a link between maternal diet and infant gender: the first component gave a good description of women's nutritional intakes in a single variable. We showed that the relationship between the scores on this variable and offspring sex differed with time period, and then we presented a further test demonstrating a link for the preconception data.

Young *et al*. maintain that because we measured women's diets at three time points, we inflated the chance of obtaining a positive result. Yet only one of these time points—the time around conception—was biologically relevant to the primary hypothesis. At the time around conception, it is possible that the maternal environment differentially favours the survival of X- or Y-bearing sperm, or differentially maintains the newly fertilized male or female embryo. Beyond this point, it would not be possible for maternal diet to affect infant gender (except by causing miscarriage or stillbirth, very rare events in the target cohort). The data from the later time frames were reported for completeness, allowing the reader to compare the findings with other studies, including some published in this journal, on maternal diet or body mass index *during* pregnancy in relation to infant gender. It is biologically implausible that the inclusion of these data provided us with a threefold greater chance of the primary hypothesis being correct.

Young *et al*. ignore the clear hierarchical structure of our analysis and interpretation. Having established the link between infant gender and maternal nutritional status around the time of conception, we went on to examine whether preconceptional energy intake—a subset of total nutrient status—was linked to infant sex. Energy was chosen as the first subsidiary variable to examine because it had been associated with offspring gender in a range of species. Other individual nutrients (*n*=17) were then examined (acknowledging the correlations between them), followed by exploratory analyses of foods (first of all grouped into 15 large categories, of which cereals were one, then individually). For reasons they do not explain, Young *et al*. have not applied their methodology to the primary evidence we presented (tables 1 and 2). Instead, they focus on the lowest tier of evidence, relating to individual food items. If it is to be used at all, their correction strategy should only be applied to tests within time periods because of the nutrition×time interaction. (That is to say, they misapply their own tests.) If we apply their method appropriately (using an identical software code) to the data in table 1, our conclusions are not materially altered: the adjusted *p*-values for several nutrients (protein, potassium, calcium) in the preconception period remain statistically significant, while none are significant at the other time points.

At each stage of our paper's analysis, the interpretation became more conservative: for example, ‘Although [potassium, calcium and sodium] did show highly significant associations with foetal sex in our study, we are cautious in the interpretation of the data until further data are available’. We had no *a priori* hypotheses concerning individual food items and were therefore cautious in the interpretation of those results. The breakfast cereal result, which Young *et al*. highlight, was not mentioned in our abstract. We also drew attention to the potential for non-nutritional factors correlated with nutrition to be influential: ‘Various non-nutritional factors have been associated with sex allocation in humans, and these may act in concert with nutritional factors or may be confounded with them’. Our discussion ends with a call for further research using biomarkers of nutritional status.

Young *et al*. make several passing remarks about other aspects of our methodology. These seem to relate to their doubts (reported elsewhere and in correspondence with us) about the use of observational studies generally, on the assumption that they all include bias, and that in high-powered (large) studies such bias will generate spurious significant results. Bias in our study could only have been introduced if women bearing male and female infants differentially misreported their diets. However, the participants did not know the gender of their infant at the time of reporting. No plausible mechanism for the introduction of bias has been proposed by Young *et al*.

Young *et al*.'s mistaken critique of our study illustrates what can go wrong when statistics are divorced from the relevant biological knowledge. Young *et al*. advocate the use of an automated procedure to adjust for multiple testing, uninformed by prior scientific knowledge. Apparently, confused about the questions being asked of the data, they employ a method that treats all variables as if they are of equal importance. This is as misguided as the blind application of stepwise regression. Virtually all observational studies, whether in ecology or epidemiology, include a range of variables, in addition to those of primary interest. These must be analysed and interpreted appropriately, but the argument that exploratory analyses should not be reported does not bear scrutiny. We ought to be thankful, after all, that Richard Doll collected ancillary data on smoking in his study of the link between lung cancer and motor vehicles.

*Addendum*. We have noted two transcriptional errors in our manuscript, neither of which alters the interpretation of the results. First, two rows in table 1 are transposed: the factor loadings for carbohydrate are those for given vitamin C and vice versa. Therefore factor 1 described diets high in a range of nutrients including carbohydrate. This is consistent with our subsequent results showing significant associations between sex ratio and peri-conceptional intakes of carbohydrate. Second, the *p*-value for the relationship between factor 1 scores and foetal sex should read 0.0095 not 0.00095.
